# Supplementary material for: Inflammatory Changes after Medical Suppression of Suspected Endometriosis for Implantation Failure: Preliminary Results
Source: Int J Mol Sci. 2024 Jun 22;25(13):6852. doi: 10.3390/ijms25136852 (PMC11241468; doi:10.3390/ijms25136852)
Supplement: Supplementary file 1 [file ijms-25-06852-s001.zip › Supplementary Table S1.pdf]

Table S1. nCounter Human Inflammation Panel gene list.

Symbol	Accession	Gene Name
AGER	NM_001136.3	advanced glycosylation end product-specific receptor
ALOX12	NM_000697.1	arachidonate 12-lipoxygenase
ALOX15	NM_001140.3	arachidonate 15-lipoxygenase
ALOX5	NM_000698.2	arachidonate 5-lipoxygenase
AREG	NM_001657.2	amphiregulin
ARG1	NM_000045.2	arginase, liver
ATF2	NM_001880.2	activating transcription factor 2
BCL2L1	NM_138578.1	BCL2-like 1
BCL6	NM_001706.2	B-cell CLL/lymphoma 6
BIRC2	NM_001166.3	baculoviral IAP repeat containing 2
C1QA	NM_015991.2	complement component 1, q subcomponent, A chain
C1QB	NM_000491.3	complement component 1, q subcomponent, B chain
C1R	NM_001733.4	complement component 1, r subcomponent
C1S	NM_001734.2	complement component 1, s subcomponent
C2	NM_000063.3	complement component 2
C3	NM_000064.2	complement component 3
C3AR1	NM_004054.2	complement component 3a receptor 1
C4A	NM_007293.2	complement component 4A (Rodgers blood group)
C5	NM_001735.2	complement component 5
C6	NM_000065.2	complement component 6
C7	NM_000587.2	complement component 7
C8A	NM_000562.2	complement component 8, alpha polypeptide
C8B	NM_000066.2	complement component 8, beta polypeptide
C9	NM_001737.3	complement component 9
CCL11	NM_002986.2	chemokine (C-C motif) ligand 11
CCL13	NM_005408.2	chemokine (C-C motif) ligand 13
CCL16	NM_004590.2	chemokine (C-C motif) ligand 16
CCL17	NM_002987.2	chemokine (C-C motif) ligand 17
CCL19	NM_006274.2	chemokine (C-C motif) ligand 19
CCL2	NM_002982.3	chemokine (C-C motif) ligand 2
CCL20	NM_004591.1	chemokine (C-C motif) ligand 20
CCL21	NM_002989.2	chemokine (C-C motif) ligand 21
CCL22	NM_002990.3	chemokine (C-C motif) ligand 22
CCL23	NM_145898.1	chemokine (C-C motif) ligand 23
CCL24	NM_002991.2	chemokine (C-C motif) ligand 24
CCL3	NM_002983.2	chemokine (C-C motif) ligand 3
CCL4	NM_002984.2	chemokine (C-C motif) ligand 4
CCL5	NM_002985.2	chemokine (C-C motif) ligand 5

CCL7	NM_006273.2	chemokine (C-C motif) ligand 7
CCL8	NM_005623.2	chemokine (C-C motif) ligand 8
CCR1	NM_001295.2	chemokine (C-C motif) receptor 1
CCR2	NM_001123041.2	chemokine (C-C motif) receptor 2
CCR3	NM_001837.2	chemokine (C-C motif) receptor 3
CCR4	NM_005508.4	chemokine (C-C motif) receptor 4
CCR7	NM_001838.2	chemokine (C-C motif) receptor 7
CD163	NM_004244.4	CD163 molecule
CD4	NM_000616.3	CD4 molecule
CD40	NM_001250.4	CD40 molecule, TNF receptor superfamily member 5
CD40LG	NM_000074.2	CD40 ligand
CD55	NM_000574.3	CD55 molecule, decay accelerating factor for complement (Cromer blood group)
CD86	NM_175862.3	CD86 molecule
CDC42	NM_001039802.1	cell division cycle 42 (GTP binding protein, 25kDa)
CEBPB	NM_005194.2	CCAAT/enhancer binding protein (C/EBP), beta
CFB	NM_001710.5	complement factor B
CFD	NM_001928.2	complement factor D (adipsin)
CFL1	NM_005507.2	cofilin 1 (non-muscle)
CREB1	NM_134442.2	cAMP responsive element binding protein 1
CRP	NM_000567.2	C-reactive protein, pentraxin-related
CSF1	NM_000757.4	colony stimulating factor 1 (macrophage)
CSF2	NM_000758.2	colony stimulating factor 2 (granulocyte-macrophage)
CSF3	NM_000759.2	colony stimulating factor 3 (granulocyte)
CXCL1	NM_001511.1	chemokine (C-X-C motif) ligand 1 (melanoma growth stimulating activity, alpha)
CXCL10	NM_001565.1	chemokine (C-X-C motif) ligand 10
CXCL2	NM_002089.3	chemokine (C-X-C motif) ligand 2
CXCL3	NM_002090.2	chemokine (C-X-C motif) ligand 3
CXCL5	NM_002994.3	chemokine (C-X-C motif) ligand 5
CXCL6	NM_002993.3	chemokine (C-X-C motif) ligand 6 (granulocyte chemotactic protein 2)
CXCL9	NM_002416.1	chemokine (C-X-C motif) ligand 9
CXCR1	NM_000634.2	chemokine (C-X-C motif) receptor 1
CXCR2	NM_001557.2	chemokine (C-X-C motif) receptor 2
CXCR4	NM_003467.2	chemokine (C-X-C motif) receptor 4
CYSLTR1	NM_006639.2	cysteinyl leukotriene receptor 1
CYSLTR2	NM_020377.2	cysteinyl leukotriene receptor 2
DAXX	NM_001350.3	death-domain associated protein
DDIT3	NM_004083.4	DNA-damage-inducible transcript 3
DEFA1	NM_004084.2	defensin, alpha 1
ELK1	NM_005229.3	ELK1, member of ETS oncogene family

FASLG	NM_000639.1	Fas ligand (TNF superfamily, member 6)
FLT1	NM_002019.4	fms-related tyrosine kinase 1 (vascular endothelial growth factor/vascular permeability factor receptor)
FOS	NM_005252.2	FBJ murine osteosarcoma viral oncogene homolog
FXYD2	NM_021603.3	FXYD domain containing ion transport regulator 2
GNAQ	NM_002072.2	guanine nucleotide binding protein (G protein), q polypeptide
GNAS	NM_080425.1	GNAS complex locus
GNB1	NM_002074.3	guanine nucleotide binding protein (G protein), beta polypeptide 1
GNGT1	NM_021955.3	guanine nucleotide binding protein (G protein), gamma transducing activity polypeptide 1
GRB2	NM_203506.2	growth factor receptor-bound protein 2
HDAC4	NM_006037.3	histone deacetylase 4
HIF1A	NM_001530.2	hypoxia inducible factor 1, alpha subunit (basic helix-loop-helix transcription factor)
HLA-DRA	NM_019111.3	major histocompatibility complex, class II, DR alpha
HLA-DRB1	NM_002124.1	major histocompatibility complex, class II, DR beta 1
HMGB1	NM_002128.4	high mobility group box 1
HMGB2	NM_001130688.1	high mobility group box 2
HMGN1	NM_004965.6	high mobility group nucleosome binding domain 1
HRAS	NM_005343.2	v-Ha-ras Harvey rat sarcoma viral oncogene homolog
HSH2D	NM_032855.2	hematopoietic SH2 domain containing
HSPB1	NM_001540.3	heat shock 27kDa protein 1
HSPB2	NM_001541.3	heat shock 27kDa protein 2
IFI44	NM_006417.4	interferon-induced protein 44
IFIT1	NM_001548.3	interferon-induced protein with tetratricopeptide repeats 1
IFIT2	NM_001547.4	interferon-induced protein with tetratricopeptide repeats 2
IFIT3	NM_001031683.2	interferon-induced protein with tetratricopeptide repeats 3
IFNA1	NM_024013.1	interferon, alpha 1
IFNB1	NM_002176.2	interferon, beta 1, fibroblast
IFNG	NM_000619.2	interferon, gamma
IL10	NM_000572.2	interleukin 10
IL10RB	NM_000628.3	interleukin 10 receptor, beta
IL11	NM_000641.2	interleukin 11
IL12A	NM_000882.2	interleukin 12A (natural killer cell stimulatory factor 1, cytotoxic lymphocyte maturation factor 1, p35)
IL12B	NM_002187.2	interleukin 12B (natural killer cell stimulatory factor 2, cytotoxic lymphocyte maturation factor 2, p40)
IL13	NM_002188.2	interleukin 13
IL15	NM_000585.3	interleukin 15
IL17A	NM_002190.2	interleukin 17A
IL18	NM_001562.2	interleukin 18 (interferon-gamma-inducing factor)
IL18RAP	NM_003853.2	interleukin 18 receptor accessory protein

IL1A	NM_000575.3	interleukin 1, alpha
IL1B	NM_000576.2	interleukin 1, beta
IL1R1	NM_000877.2	interleukin 1 receptor, type I
IL1RAP	NM_002182.2	interleukin 1 receptor accessory protein
IL1RN	NM_173842.1	interleukin 1 receptor antagonist
IL2	NM_000586.2	interleukin 2
IL21	NM_021803.2	interleukin 21
IL22	NM_020525.4	interleukin 22
IL22RA2	NM_181309.1	interleukin 22 receptor, alpha 2
IL23A	NM_016584.2	interleukin 23, alpha subunit p19
IL23R	NM_144701.2	interleukin 23 receptor
IL3	NM_000588.3	interleukin 3 (colony-stimulating factor, multiple)
IL4	NM_000589.2	interleukin 4
IL5	NM_000879.2	interleukin 5 (colony-stimulating factor, eosinophil)
IL6	NM_000600.1	interleukin 6 (interferon, beta 2)
IL6R	NM_000565.2	interleukin 6 receptor
IL7	NM_000880.2	interleukin 7
IL8	NM_000584.2	interleukin 8
IL9	NM_000590.1	interleukin 9
IRF1	NM_002198.1	interferon regulatory factor 1
IRF3	NM_001571.5	interferon regulatory factor 3
IRF5	NM_002200.3	interferon regulatory factor 5
IRF7	NM_001572.3	interferon regulatory factor 7
ITGB2	NM_000211.2	integrin, beta 2 (complement component 3 receptor 3 and 4 subunit)
JUN	NM_002228.3	jun proto-oncogene
KEAP1	NM_012289.3	kelch-like ECH-associated protein 1
KNG1	NM_000893.2	kininogen 1
LIMK1	NM_002314.3	LIM domain kinase 1
LTA	NM_000595.2	lymphotoxin alpha (TNF superfamily, member 1)
LTB	NM_002341.1	lymphotoxin beta (TNF superfamily, member 3)
LTB4R	NM_181657.3	leukotriene B4 receptor
LTB4R2	NM_019839.4	leukotriene B4 receptor 2
LY96	NM_015364.2	lymphocyte antigen 96
MAFF	NM_001161572.1	v-maf musculoaponeurotic fibrosarcoma oncogene homolog F (avian)
MAFG	NM_002359.2	v-maf musculoaponeurotic fibrosarcoma oncogene homolog G (avian)
MAFK	NM_002360.3	v-maf musculoaponeurotic fibrosarcoma oncogene homolog K (avian)
MAP2K1	NM_002755.2	mitogen-activated protein kinase kinase 1
MAP2K4	NM_003010.2	mitogen-activated protein kinase kinase 4
MAP2K6	NM_002758.3	mitogen-activated protein kinase kinase 6

MAP3K1	NM_005921.1	mitogen-activated protein kinase kinase kinase 1, E3 ubiquitin protein ligase
MAP3K5	NM_005923.3	mitogen-activated protein kinase kinase kinase 5
MAP3K7	NM_145333.1	mitogen-activated protein kinase kinase kinase 7
MAP3K9	NM_033141.2	mitogen-activated protein kinase kinase kinase 9
MAPK1	NM_138957.2	mitogen-activated protein kinase 1
MAPK14	NM_001315.1	mitogen-activated protein kinase 14
MAPK3	NM_001040056.1	mitogen-activated protein kinase 3
MAPK8	NM_002750.2	mitogen-activated protein kinase 8
MAPKAPK2	NM_004759.3	mitogen-activated protein kinase-activated protein kinase 2
MAPKAPK5	NM_003668.2	mitogen-activated protein kinase-activated protein kinase 5
MASP1	NM_139125.3	mannan-binding lectin serine peptidase 1 (C4/C2 activating component of Ra-reactive factor)
MASP2	NM_139208.1	mannan-binding lectin serine peptidase 2
MAX	NM_002382.3	MYC associated factor X
MBL2	NM_000242.2	mannose-binding lectin (protein C) 2, soluble
MEF2A	NM_005587.2	myocyte enhancer factor 2A
MEF2BNB-MEF2B	NM_005919.2	MEF2BNB-MEF2B readthrough
MEF2C	NM_002397.3	myocyte enhancer factor 2C
MEF2D	NM_005920.2	myocyte enhancer factor 2D
MKNK1	NM_003684.3	MAP kinase interacting serine/threonine kinase 1
MMP3	NM_002422.3	matrix metallopeptidase 3 (stromelysin 1, progelatinase)
MMP9	NM_004994.2	matrix metallopeptidase 9 (gelatinase B, 92kDa gelatinase, 92kDa type IV collagenase)
MRC1	NM_002438.2	mannose receptor, C type 1
MX1	NM_002462.2	myxovirus (influenza virus) resistance 1, interferon-inducible protein p78 (mouse)
MX2	NM_002463.1	myxovirus (influenza virus) resistance 2 (mouse)
MYC	NM_002467.3	v-myc myelocytomatosis viral oncogene homolog (avian)
MYD88	NM_002468.3	myeloid differentiation primary response gene (88)
MYL2	NM_000432.3	myosin, light chain 2, regulatory, cardiac, slow
NFATC3	NM_004555.2	nuclear factor of activated T-cells, cytoplasmic, calcineurin-dependent 3
NFE2L2	NM_006164.3	nuclear factor (erythroid-derived 2)-like 2
NFKB1	NM_003998.2	nuclear factor of kappa light polypeptide gene enhancer in B-cells 1
NLRP3	NM_001079821.2	NLR family, pyrin domain containing 3
NOD1	NM_006092.1	nucleotide-binding oligomerization domain containing 1
NOD2	NM_022162.1	nucleotide-binding oligomerization domain containing 2
NOS2	NM_000625.4	nitric oxide synthase 2, inducible
NOX1	NM_007052.4	NADPH oxidase 1

NR3C1	NM_001018074.1	nuclear receptor subfamily 3, group C, member 1 (glucocorticoid receptor)
OAS2	NM_016817.2	2'-5'-oligoadenylate synthetase 2, 69/71kDa
OASL	NM_198213.1	2'-5'-oligoadenylate synthetase-like
OXER1	NM_148962.3	oxoeicosanoid (OXE) receptor 1
PDGFA	NM_002607.5	platelet-derived growth factor alpha polypeptide
PIK3C2G	NM_004570.4	phosphatidylinositol-4-phosphate 3-kinase, catalytic subunit type 2 gamma
PLA2G4A	NM_024420.2	phospholipase A2, group IVA (cytosolic, calcium-dependent)
PLCB1	NM_182734.1	phospholipase C, beta 1 (phosphoinositide-specific)
PPP1R12B	NM_002481.3	protein phosphatase 1, regulatory subunit 12B
PRKCA	NM_002737.2	protein kinase C, alpha
PRKCB	NM_212535.1	protein kinase C, beta
PTGDR2	NM_004778.1	prostaglandin D2 receptor 2
PTGER1	NM_000955.2	prostaglandin E receptor 1 (subtype EP1), 42kDa
PTGER2	NM_000956.2	prostaglandin E receptor 2 (subtype EP2), 53kDa
PTGER3	NM_000957.2	prostaglandin E receptor 3 (subtype EP3)
PTGER4	NM_000958.2	prostaglandin E receptor 4 (subtype EP4)
PTGFR	NM_000959.3	prostaglandin F receptor (FP)
PTGIR	NM_000960.3	prostaglandin I2 (prostacyclin) receptor (IP)
PTGS1	NM_000962.2	prostaglandin-endoperoxide synthase 1 (prostaglandin G/H synthase and cyclooxygenase)
PTGS2	NM_000963.1	prostaglandin-endoperoxide synthase 2 (prostaglandin G/H synthase and cyclooxygenase)
PTK2	NM_005607.3	PTK2 protein tyrosine kinase 2
RAC1	NM_198829.1	ras-related C3 botulinum toxin substrate 1 (rho family, small GTP binding protein Rac1)
RAF1	NM_002880.2	v-raf-1 murine leukemia viral oncogene homolog 1
RAPGEF2	NM_014247.2	Rap guanine nucleotide exchange factor (GEF) 2
RELA	NM_021975.2	v-rel reticuloendotheliosis viral oncogene homolog A (avian)
RELB	NM_006509.2	v-rel reticuloendotheliosis viral oncogene homolog B
RHOA	NM_001664.2	ras homolog family member A
RIPK1	NM_003804.3	receptor (TNFRSF)-interacting serine-threonine kinase 1
RIPK2	NM_003821.5	receptor-interacting serine-threonine kinase 2
ROCK2	NM_004850.3	Rho-associated, coiled-coil containing protein kinase 2
RPS6KA5	NM_004755.2	ribosomal protein S6 kinase, 90kDa, polypeptide 5
SHC1	NM_001130040.1	SHC (Src homology 2 domain containing) transforming protein 1
SMAD7	NM_005904.2	SMAD family member 7
STAT1	NM_007315.2	signal transducer and activator of transcription 1, 91kDa
STAT2	NM_005419.2	signal transducer and activator of transcription 2, 113kDa
STAT3	NM_139276.2	signal transducer and activator of transcription 3 (acute-phase response factor)

TBXA2R	NM_001060.3	thromboxane A2 receptor
TCF4	NM_003199.1	transcription factor 4
TGFB1	NM_000660.3	transforming growth factor, beta 1
TGFB2	NM_003238.2	transforming growth factor, beta 2
TGFB3	NM_003239.2	transforming growth factor, beta 3
TGFBR1	NM_004612.2	transforming growth factor, beta receptor 1
TLR1	NM_003263.3	toll-like receptor 1
TLR2	NM_003264.3	toll-like receptor 2
TLR3	NM_003265.2	toll-like receptor 3
TLR4	NM_138554.2	toll-like receptor 4
TLR5	NM_003268.3	toll-like receptor 5
TLR6	NM_006068.2	toll-like receptor 6
TLR7	NM_016562.3	toll-like receptor 7
TLR8	NM_016610.2	toll-like receptor 8
TLR9	NM_017442.2	toll-like receptor 9
TNF	NM_000594.2	tumor necrosis factor
TNFAIP3	NM_006290.2	tumor necrosis factor, alpha-induced protein 3
TNFSF14	NM_003807.2	tumor necrosis factor (ligand) superfamily, member 14
TOLLIP	NM_019009.2	toll interacting protein
TRADD	NM_003789.2	TNFRSF1A-associated via death domain
TRAF2	NM_021138.3	TNF receptor-associated factor 2
TREM2	NM_018965.3	triggering receptor expressed on myeloid cells 2
TSLP	NM_033035.4	thymic stromal lymphopoietin
TWIST2	NM_057179.2	twist homolog 2 (Drosophila)
TYROBP	NM_003332.3	TYRO protein tyrosine kinase binding protein
**Internal Reference Genes**		
CLTC	NM_004859.2	clathrin, heavy chain (Hc)
GAPDH	NM_002046.3	glyceraldehyde-3-phosphate dehydrogenase
GUSB	NM_000181.1	glucuronidase, beta
HPRT1	NM_000194.1	hypoxanthine phosphoribosyltransferase 1
PGK1	NM_000291.2	phosphoglycerate kinase 1
TUBB	NM_178014.2	tubulin, beta class I
