# Supplementary material for: Inflammatory Changes after Medical Suppression of Suspected Endometriosis for Implantation Failure: Preliminary Results
Source: Int J Mol Sci. 2024 Jun 22;25(13):6852. doi: 10.3390/ijms25136852 (PMC11241468; doi:10.3390/ijms25136852)
Supplement: Supplementary file 1 [file ijms-25-06852-s001.zip › Supplementary Table S2.pdf]

Table S2. DETs in pre- and post-treatment comparison following treatment with OCPs.

Symbol	Accession	p-value	Fold change
TGFB1	NM_000660.3	0.035516068	1.064399379
IL10RB	NM_000628.3	0.047698183	1.24448641
MX2	NM_002463.1	0.045870139	1.206619117
GNGT1	NM_021955.3	0.007194072	0.334312887
CCL19	NM_006274.2	0.002886782	0.339778991
MASP2	NM_139208.1	0.03458245	0.338439139
CCL2	NM_002982.3	0.037994478	0.367985367
IL12B	NM_002187.2	0.041153509	0.330361887
CCL11	NM_002986.2	0.012966004	0.288807563
NOX1	NM_007052.4	0.036819682	0.42015773
