# Supplementary material for: Inflammatory Changes after Medical Suppression of Suspected Endometriosis for Implantation Failure: Preliminary Results
Source: Int J Mol Sci. 2024 Jun 22;25(13):6852. doi: 10.3390/ijms25136852 (PMC11241468; doi:10.3390/ijms25136852)
Supplement: Supplementary file 1 [file ijms-25-06852-s001.zip › Supplementary Table S3.pdf]

Table S3. DETs in pre- and post-treatment comparison following treatment with elagolix.

Symbol	Accession	p-value	fold change
HSPB2	NM_001541.3	0.000557425	3.900644969
CCL22	NM_002990.3	0.001592407	5.356634559
C1QA	NM_015991.2	0.036624055	2.537477648
CFB	NM_001710.5	0.002029014	3.259175663
C9	NM_001737.3	0.033423633	3.288809167
TWIST2	NM_057179.2	0.025961545	2.734267874
NOS2	NM_000625.4	0.003831234	2.13423301
IL22RA2	NM_181309.1	0.003782793	2.476295001
CSF3	NM_000759.2	0.008822947	3.328305131
CCL2	NM_002982.3	0.005379109	3.879485632
IL17A	NM_002190.2	0.008161233	2.147837385
CCL8	NM_005623.2	0.007673625	4.149870319
CCL24	NM_002991.2	0.006343891	2.375496607
MASP1	NM_139125.3	0.026937238	2.601558544
MYL2	NM_000432.3	0.012195594	3.71130796
C8B	NM_000066.2	0.021343861	2.281933257
MMP3	NM_002422.3	0.000844241	4.474195345
HSPB1	NM_001540.3	0.021620025	1.736043036
CXCL10	NM_001565.1	0.00943428	3.254040578
PDGFA	NM_002607.5	0.002199318	2.532692238
CSF2	NM_000758.2	0.032746562	2.420765718
IL21	NM_021803.2	0.008063651	3.098356144
CCL19	NM_006274.2	0.029168293	2.257266503
FLT1	NM_002019.4	0.040539322	1.477034831
IL11	NM_000641.2	0.000230026	3.630813113
CCL17	NM_002987.2	0.013658795	3.680476267
IFNG	NM_000619.2	0.017151104	2.089378165
IL13	NM_002188.2	0.006836528	2.04627476
IFNB1	NM_002176.2	0.012343131	3.333441999
HRAS	NM_005343.2	0.011226105	1.77864082
CCL3	NM_002983.2	0.027071252	1.600419561
CCL7	NM_006273.2	0.010468777	2.603764166
TLR9	NM_017442.2	0.009306338	1.497988541
C1R	NM_001733.4	0.009703125	2.84328098
TRADD	NM_003789.2	0.033101042	1.380089323
MAP3K9	NM_033141.2	0.014455324	1.952399902
TGFB3	NM_003239.2	0.009331222	1.815408132
TGFB2	NM_003238.2	0.004188691	2.662891322
PTGDR2	NM_004778.1	0.004727948	1.777357662
CCL4	NM_002984.2	0.049136689	1.383796535
ALOX15	NM_001140.3	0.030587356	2.388876117
NOD1	NM_006092.1	0.025418528	1.567685045
