# Supplementary material for: Inflammatory Changes after Medical Suppression of Suspected Endometriosis for Implantation Failure: Preliminary Results
Source: Int J Mol Sci. 2024 Jun 22;25(13):6852. doi: 10.3390/ijms25136852 (PMC11241468; doi:10.3390/ijms25136852)
Supplement: Supplementary file 1 [file ijms-25-06852-s001.zip › Supplementary Table S4.pdf]

Table S4. nCounter Human miRNA Panel gene list

**Symbol**	**Accession**
hsa-let-7a-5p	MIMAT0000062
hsa-let-7b-5p	MIMAT0000063
hsa-let-7c-5p	MIMAT0000064
hsa-let-7d-5p	MIMAT0000065
hsa-let-7e-5p	MIMAT0000066
hsa-let-7f-5p	MIMAT0000067
hsa-let-7g-5p	MIMAT0000414
hsa-let-7i-5p	MIMAT0000415
hsa-miR-1-3p	MIMAT0000416
hsa-miR-1-5p	MIMAT0031892
hsa-miR-100-5p	MIMAT0000098
hsa-miR-101-3p	MIMAT0000099
hsa-miR-103a-3p	MIMAT0000101
hsa-miR-105-5p	MIMAT0000102
hsa-miR-106a-5p+hsa-miR-17-5p	MIMAT0000103
hsa-miR-106b-5p	MIMAT0000680
hsa-miR-107	MIMAT0000104
hsa-miR-10a-5p	MIMAT0000253
hsa-miR-10b-5p	MIMAT0000254
hsa-miR-1178-3p	MIMAT0005823
hsa-miR-1180-3p	MIMAT0005825
hsa-miR-1183	MIMAT0005828
hsa-miR-1185-1-3p	MIMAT0022838
hsa-miR-1185-2-3p	MIMAT0022713
hsa-miR-1185-5p	MIMAT0005798
hsa-miR-1193	MIMAT0015049
hsa-miR-1197	MIMAT0005955
hsa-miR-1200	MIMAT0005863
hsa-miR-1202	MIMAT0005865
hsa-miR-1203	MIMAT0005866
hsa-miR-1204	MIMAT0005868
hsa-miR-1205	MIMAT0005869
hsa-miR-1206	MIMAT0005870
hsa-miR-122-5p	MIMAT0000421
hsa-miR-1224-3p	MIMAT0005459
hsa-miR-1224-5p	MIMAT0005458
hsa-miR-1226-3p	MIMAT0005577
hsa-miR-1228-3p	MIMAT0005583
hsa-miR-1233-3p	MIMAT0005588
hsa-miR-1234-3p	MIMAT0005589
hsa-miR-1236-3p	MIMAT0005591
hsa-miR-124-3p	MIMAT0000422
hsa-miR-1244	MIMAT0005896
hsa-miR-1245a	MIMAT0005897

hsa-miR-1245b-3p	MIMAT0019951
hsa-miR-1245b-5p	MIMAT0019950
hsa-miR-1246	MIMAT0005898
hsa-miR-1247-5p	MIMAT0005899
hsa-miR-1248	MIMAT0005900
hsa-miR-1249-3p	MIMAT0005901
hsa-miR-1249-5p	MIMAT0032029
hsa-miR-1250-5p	MIMAT0005902
hsa-miR-1252-5p	MIMAT0005944
hsa-miR-1253	MIMAT0005904
hsa-miR-1254	MIMAT0005905
hsa-miR-1255a	MIMAT0005906
hsa-miR-1255b-5p	MIMAT0005945
hsa-miR-1257	MIMAT0005908
hsa-miR-1258	MIMAT0005909
hsa-miR-125a-3p	MIMAT0004602
hsa-miR-125a-5p	MIMAT0000443
hsa-miR-125b-5p	MIMAT0000423
hsa-miR-126-3p	MIMAT0000445
hsa-miR-1260a	MIMAT0005911
hsa-miR-1260b	MIMAT0015041
hsa-miR-1261	MIMAT0005913
hsa-miR-1262	MIMAT0005914
hsa-miR-1264	MIMAT0005791
hsa-miR-1266-5p	MIMAT0005920
hsa-miR-1268a	MIMAT0005922
hsa-miR-1268b	MIMAT0018925
hsa-miR-1269a	MIMAT0005923
hsa-miR-1269b	MIMAT0019059
hsa-miR-127-3p	MIMAT0000446
hsa-miR-127-5p	MIMAT0004604
hsa-miR-1270	MIMAT0005924
hsa-miR-1271-3p	MIMAT0022712
hsa-miR-1271-5p	MIMAT0005796
hsa-miR-1272	MIMAT0005925
hsa-miR-1273c	MIMAT0015017
hsa-miR-1275	MIMAT0005929
hsa-miR-1276	MIMAT0005930
hsa-miR-1277-3p	MIMAT0005933
hsa-miR-1278	MIMAT0005936
hsa-miR-1279	MIMAT0005937
hsa-miR-128-1-5p	MIMAT0026477
hsa-miR-128-2-5p	MIMAT0031095
hsa-miR-128-3p	MIMAT0000424
hsa-miR-1281	MIMAT0005939
hsa-miR-1283	MIMAT0005799
hsa-miR-1285-3p	MIMAT0005876

hsa-miR-1285-5p	MIMAT0022719
hsa-miR-1286	MIMAT0005877
hsa-miR-1287-3p	MIMAT0026738
hsa-miR-1287-5p	MIMAT0005878
hsa-miR-1288-3p	MIMAT0005942
hsa-miR-1289	MIMAT0005879
hsa-miR-129-2-3p	MIMAT0004605
hsa-miR-129-5p	MIMAT0000242
hsa-miR-1290	MIMAT0005880
hsa-miR-1291	MIMAT0005881
hsa-miR-1293	MIMAT0005883
hsa-miR-1295a	MIMAT0005885
hsa-miR-1296-3p	MIMAT0026637
hsa-miR-1296-5p	MIMAT0005794
hsa-miR-1297	MIMAT0005886
hsa-miR-1298-5p	MIMAT0005800
hsa-miR-1299	MIMAT0005887
hsa-miR-1301-3p	MIMAT0005797
hsa-miR-1302	MIMAT0005890
hsa-miR-1303	MIMAT0005891
hsa-miR-1304-3p	MIMAT0022720
hsa-miR-1304-5p	MIMAT0005892
hsa-miR-1305	MIMAT0005893
hsa-miR-1306-3p	MIMAT0005950
hsa-miR-1306-5p	MIMAT0022726
hsa-miR-1307-3p	MIMAT0005951
hsa-miR-1307-5p	MIMAT0022727
hsa-miR-130a-3p	MIMAT0000425
hsa-miR-130b-3p	MIMAT0000691
hsa-miR-132-3p	MIMAT0000426
hsa-miR-1322	MIMAT0005953
hsa-miR-1323	MIMAT0005795
hsa-miR-133a-3p	MIMAT0000427
hsa-miR-133a-5p	MIMAT0026478
hsa-miR-133b	MIMAT0000770
hsa-miR-134-3p	MIMAT0026481
hsa-miR-134-5p+hsa-miR-6728-5p	MIMAT0000447
hsa-miR-135a-5p	MIMAT0000428
hsa-miR-135b-5p	MIMAT0000758
hsa-miR-136-5p	MIMAT0000448
hsa-miR-137	MIMAT0000429
hsa-miR-138-5p	MIMAT0000430
hsa-miR-139-3p	MIMAT0004552
hsa-miR-139-5p	MIMAT0000250
hsa-miR-140-3p	MIMAT0004597
hsa-miR-140-5p	MIMAT0000431
hsa-miR-141-3p	MIMAT0000432

hsa-miR-142-3p	MIMAT0000434
hsa-miR-142-5p	MIMAT0000433
hsa-miR-143-3p	MIMAT0000435
hsa-miR-144-3p	MIMAT0000436
hsa-miR-145-5p	MIMAT0000437
hsa-miR-1469	MIMAT0007347
hsa-miR-146a-5p	MIMAT0000449
hsa-miR-146b-3p	MIMAT0004766
hsa-miR-146b-5p	MIMAT0002809
hsa-miR-147a	MIMAT0000251
hsa-miR-147b	MIMAT0004928
hsa-miR-148a-3p	MIMAT0000243
hsa-miR-148b-3p	MIMAT0000759
hsa-miR-149-5p	MIMAT0000450
hsa-miR-150-5p	MIMAT0000451
hsa-miR-151a-3p	MIMAT0000757
hsa-miR-151a-5p	MIMAT0004697
hsa-miR-151b	MIMAT0010214
hsa-miR-152-3p	MIMAT0000438
hsa-miR-152-5p	MIMAT0026479
hsa-miR-153-3p	MIMAT0000439
hsa-miR-1537-3p	MIMAT0007399
hsa-miR-154-5p	MIMAT0000452
hsa-miR-155-5p	MIMAT0000646
hsa-miR-15a-5p	MIMAT0000068
hsa-miR-15b-5p	MIMAT0000417
hsa-miR-16-5p	MIMAT0000069
hsa-miR-181a-2-3p	MIMAT0004558
hsa-miR-181a-3p	MIMAT0000270
hsa-miR-181a-5p	MIMAT0000256
hsa-miR-181b-2-3p	MIMAT0031893
hsa-miR-181b-5p+hsa-miR-181d-5	MIMAT0000257
hsa-miR-181c-5p	MIMAT0000258
hsa-miR-181d-3p	MIMAT0026608
hsa-miR-182-3p	MIMAT0000260
hsa-miR-182-5p	MIMAT0000259
hsa-miR-1827	MIMAT0006767
hsa-miR-183-5p	MIMAT0000261
hsa-miR-184	MIMAT0000454
hsa-miR-185-5p	MIMAT0000455
hsa-miR-186-5p	MIMAT0000456
hsa-miR-187-3p	MIMAT0000262
hsa-miR-188-3p	MIMAT0004613
hsa-miR-188-5p	MIMAT0000457
hsa-miR-18a-5p	MIMAT0000072
hsa-miR-18b-5p	MIMAT0001412
hsa-miR-1908-3p	MIMAT0026916

hsa-miR-1908-5p	MIMAT0007881
hsa-miR-1909-3p	MIMAT0007883
hsa-miR-190a-3p	MIMAT0026482
hsa-miR-190a-5p	MIMAT0000458
hsa-miR-190b	MIMAT0004929
hsa-miR-191-5p	MIMAT0000440
hsa-miR-1910-3p	MIMAT0026917
hsa-miR-1910-5p	MIMAT0007884
hsa-miR-1915-3p	MIMAT0007892
hsa-miR-192-5p	MIMAT0000222
hsa-miR-193a-3p	MIMAT0000459
hsa-miR-193a-5p+hsa-miR-193b-5	MIMAT0004614
hsa-miR-193b-3p	MIMAT0002819
hsa-miR-194-5p	MIMAT0000460
hsa-miR-195-5p	MIMAT0000461
hsa-miR-196a-3p	MIMAT0004562
hsa-miR-196a-5p	MIMAT0000226
hsa-miR-196b-5p	MIMAT0001080
hsa-miR-197-3p	MIMAT0000227
hsa-miR-197-5p	MIMAT0022691
hsa-miR-1972	MIMAT0009447
hsa-miR-1973	MIMAT0009448
hsa-miR-1976	MIMAT0009451
hsa-miR-198	MIMAT0000228
hsa-miR-199a-3p+hsa-miR-199b-3	MIMAT0000232
hsa-miR-199a-5p	MIMAT0000231
hsa-miR-199b-5p	MIMAT0000263
hsa-miR-19a-3p	MIMAT0000073
hsa-miR-19b-3p	MIMAT0000074
hsa-miR-200a-3p	MIMAT0000682
hsa-miR-200b-3p	MIMAT0000318
hsa-miR-200c-3p	MIMAT0000617
hsa-miR-202-3p	MIMAT0002811
hsa-miR-203a-3p	MIMAT0000264
hsa-miR-203a-5p	MIMAT0031890
hsa-miR-204-5p	MIMAT0000265
hsa-miR-205-5p	MIMAT0000266
hsa-miR-2053	MIMAT0009978
hsa-miR-206	MIMAT0000462
hsa-miR-208a-3p	MIMAT0000241
hsa-miR-208b-3p	MIMAT0004960
hsa-miR-208b-5p	MIMAT0026722
hsa-miR-20a-5p+hsa-miR-20b-5p	MIMAT0000075
hsa-miR-21-5p	MIMAT0000076
hsa-miR-210-3p	MIMAT0000267
hsa-miR-210-5p	MIMAT0026475
hsa-miR-211-3p	MIMAT0022694

hsa-miR-211-5p	MIMAT0000268
hsa-miR-2110	MIMAT0010133
hsa-miR-2113	MIMAT0009206
hsa-miR-2116-5p	MIMAT0011160
hsa-miR-2117	MIMAT0011162
hsa-miR-212-3p	MIMAT0000269
hsa-miR-214-3p	MIMAT0000271
hsa-miR-215-5p	MIMAT0000272
hsa-miR-216a-5p	MIMAT0000273
hsa-miR-216b-5p	MIMAT0004959
hsa-miR-217	MIMAT0000274
hsa-miR-218-5p	MIMAT0000275
hsa-miR-219a-1-3p	MIMAT0004567
hsa-miR-219a-2-3p	MIMAT0004675
hsa-miR-219a-5p	MIMAT0000276
hsa-miR-219b-3p	MIMAT0019748
hsa-miR-22-3p	MIMAT0000077
hsa-miR-221-3p	MIMAT0000278
hsa-miR-221-5p	MIMAT0004568
hsa-miR-222-3p	MIMAT0000279
hsa-miR-223-3p	MIMAT0000280
hsa-miR-224-5p	MIMAT0000281
hsa-miR-2278	MIMAT0011778
hsa-miR-23a-3p	MIMAT0000078
hsa-miR-23b-3p	MIMAT0000418
hsa-miR-23c	MIMAT0018000
hsa-miR-24-3p	MIMAT0000080
hsa-miR-25-3p	MIMAT0000081
hsa-miR-25-5p	MIMAT0004498
hsa-miR-2682-5p	MIMAT0013517
hsa-miR-26a-5p	MIMAT0000082
hsa-miR-26b-5p	MIMAT0000083
hsa-miR-27a-3p	MIMAT0000084
hsa-miR-27b-3p	MIMAT0000419
hsa-miR-28-3p	MIMAT0004502
hsa-miR-28-5p	MIMAT0000085
hsa-miR-296-3p	MIMAT0004679
hsa-miR-296-5p	MIMAT0000690
hsa-miR-297	MIMAT0004450
hsa-miR-298	MIMAT0004901
hsa-miR-299-3p	MIMAT0000687
hsa-miR-299-5p	MIMAT0002890
hsa-miR-29a-3p	MIMAT0000086
hsa-miR-29b-3p	MIMAT0000100
hsa-miR-29c-3p	MIMAT0000681
hsa-miR-300	MIMAT0004903
hsa-miR-301a-3p	MIMAT0000688

hsa-miR-301a-5p	MIMAT0022696
hsa-miR-301b-3p	MIMAT0004958
hsa-miR-301b-5p	MIMAT0032026
hsa-miR-302a-3p	MIMAT0000684
hsa-miR-302a-5p	MIMAT0000683
hsa-miR-302b-3p	MIMAT0000715
hsa-miR-302c-3p	MIMAT0000717
hsa-miR-302d-3p	MIMAT0000718
hsa-miR-302e	MIMAT0005931
hsa-miR-302f	MIMAT0005932
hsa-miR-3065-3p	MIMAT0015378
hsa-miR-3065-5p	MIMAT0015066
hsa-miR-3074-3p	MIMAT0015027
hsa-miR-30a-3p	MIMAT0000088
hsa-miR-30a-5p	MIMAT0000087
hsa-miR-30b-5p	MIMAT0000420
hsa-miR-30c-5p	MIMAT0000244
hsa-miR-30d-5p	MIMAT0000245
hsa-miR-30e-3p	MIMAT0000693
hsa-miR-30e-5p	MIMAT0000692
hsa-miR-31-5p	MIMAT0000089
hsa-miR-3127-5p	MIMAT0014990
hsa-miR-3130-3p	MIMAT0014994
hsa-miR-3131	MIMAT0014996
hsa-miR-3136-5p	MIMAT0015003
hsa-miR-3140-3p	MIMAT0015008
hsa-miR-3140-5p	MIMAT0019204
hsa-miR-3144-3p	MIMAT0015015
hsa-miR-3144-5p	MIMAT0015014
hsa-miR-3147	MIMAT0015019
hsa-miR-3150b-3p	MIMAT0018194
hsa-miR-3151-5p	MIMAT0015024
hsa-miR-3158-3p	MIMAT0015032
hsa-miR-3161	MIMAT0015035
hsa-miR-3164	MIMAT0015038
hsa-miR-3168	MIMAT0015043
hsa-miR-3179	MIMAT0015056
hsa-miR-3180	MIMAT0018178
hsa-miR-3180-3p	MIMAT0015058
hsa-miR-3180-5p	MIMAT0015057
hsa-miR-3182	MIMAT0015062
hsa-miR-3185	MIMAT0015065
hsa-miR-3190-3p	MIMAT0022839
hsa-miR-3192-5p	MIMAT0015076
hsa-miR-3195	MIMAT0015079
hsa-miR-3196	MIMAT0015080
hsa-miR-32-5p	MIMAT0000090

hsa-miR-3202	MIMAT0015089
hsa-miR-320a	MIMAT0000510
hsa-miR-320b	MIMAT0005792
hsa-miR-320c	MIMAT0005793
hsa-miR-320d	MIMAT0006764
hsa-miR-320e	MIMAT0015072
hsa-miR-323a-3p	MIMAT0000755
hsa-miR-323a-5p	MIMAT0004696
hsa-miR-323b-3p	MIMAT0015050
hsa-miR-323b-5p	MIMAT0001630
hsa-miR-324-3p	MIMAT0000762
hsa-miR-324-5p	MIMAT0000761
hsa-miR-325	MIMAT0000771
hsa-miR-326	MIMAT0000756
hsa-miR-328-3p	MIMAT0000752
hsa-miR-328-5p	MIMAT0026486
hsa-miR-329-3p	MIMAT0001629
hsa-miR-329-5p	MIMAT0026555
hsa-miR-330-3p	MIMAT0000751
hsa-miR-330-5p	MIMAT0004693
hsa-miR-331-3p	MIMAT0000760
hsa-miR-331-5p	MIMAT0004700
hsa-miR-335-5p	MIMAT0000765
hsa-miR-337-3p	MIMAT0000754
hsa-miR-337-5p	MIMAT0004695
hsa-miR-338-5p	MIMAT0004701
hsa-miR-339-3p	MIMAT0004702
hsa-miR-339-5p	MIMAT0000764
hsa-miR-33a-5p	MIMAT0000091
hsa-miR-33b-5p	MIMAT0003301
hsa-miR-340-5p	MIMAT0004692
hsa-miR-342-3p	MIMAT0000753
hsa-miR-342-5p	MIMAT0004694
hsa-miR-345-3p	MIMAT0022698
hsa-miR-345-5p	MIMAT0000772
hsa-miR-346	MIMAT0000773
hsa-miR-34a-5p	MIMAT0000255
hsa-miR-34b-3p	MIMAT0004676
hsa-miR-34c-3p	MIMAT0004677
hsa-miR-34c-5p	MIMAT0000686
hsa-miR-3605-3p	MIMAT0017982
hsa-miR-3605-5p	MIMAT0017981
hsa-miR-361-3p	MIMAT0004682
hsa-miR-361-5p	MIMAT0000703
hsa-miR-3613-3p	MIMAT0017991
hsa-miR-3613-5p	MIMAT0017990
hsa-miR-3614-3p	MIMAT0017993

hsa-miR-3614-5p	MIMAT0017992
hsa-miR-3615	MIMAT0017994
hsa-miR-362-3p	MIMAT0004683
hsa-miR-362-5p	MIMAT0000705
hsa-miR-363-3p	MIMAT0000707
hsa-miR-363-5p	MIMAT0003385
hsa-miR-365a-3p+hsa-miR-365b-3	MIMAT0000710
hsa-miR-365b-5p	MIMAT0022833
hsa-miR-367-3p	MIMAT0000719
hsa-miR-369-3p	MIMAT0000721
hsa-miR-369-5p	MIMAT0001621
hsa-miR-3690	MIMAT0018119
hsa-miR-370-3p	MIMAT0000722
hsa-miR-370-5p	MIMAT0026483
hsa-miR-371a-5p	MIMAT0004687
hsa-miR-371b-5p	MIMAT0019892
hsa-miR-372-3p	MIMAT0000724
hsa-miR-373-3p	MIMAT0000726
hsa-miR-374a-3p	MIMAT0004688
hsa-miR-374a-5p	MIMAT0000727
hsa-miR-374b-5p	MIMAT0004955
hsa-miR-374c-5p	MIMAT0018443
hsa-miR-375	MIMAT0000728
hsa-miR-376a-2-5p	MIMAT0022928
hsa-miR-376a-3p	MIMAT0000729
hsa-miR-376b-3p	MIMAT0002172
hsa-miR-376c-3p	MIMAT0000720
hsa-miR-376c-5p	MIMAT0022861
hsa-miR-377-3p	MIMAT0000730
hsa-miR-378b	MIMAT0014999
hsa-miR-378c	MIMAT0016847
hsa-miR-378d	MIMAT0018926
hsa-miR-378e	MIMAT0018927
hsa-miR-378f	MIMAT0018932
hsa-miR-378g	MIMAT0018937
hsa-miR-378h	MIMAT0018984
hsa-miR-378i	MIMAT0019074
hsa-miR-379-5p	MIMAT0000733
hsa-miR-380-3p	MIMAT0000735
hsa-miR-381-3p	MIMAT0000736
hsa-miR-381-5p	MIMAT0022862
hsa-miR-382-3p	MIMAT0022697
hsa-miR-382-5p	MIMAT0000737
hsa-miR-383-5p	MIMAT0000738
hsa-miR-384	MIMAT0001075
hsa-miR-3916	MIMAT0018190
hsa-miR-3918	MIMAT0018192

hsa-miR-3928-3p	MIMAT0018205
hsa-miR-3934-5p	MIMAT0018349
hsa-miR-409-3p	MIMAT0001639
hsa-miR-409-5p	MIMAT0001638
hsa-miR-410-3p	MIMAT0002171
hsa-miR-411-5p	MIMAT0003329
hsa-miR-412-3p	MIMAT0002170
hsa-miR-421	MIMAT0003339
hsa-miR-422a	MIMAT0001339
hsa-miR-423-3p	MIMAT0001340
hsa-miR-423-5p	MIMAT0004748
hsa-miR-424-5p	MIMAT0001341
hsa-miR-425-5p	MIMAT0003393
hsa-miR-4284	MIMAT0016915
hsa-miR-4286	MIMAT0016916
hsa-miR-429	MIMAT0001536
hsa-miR-431-5p	MIMAT0001625
hsa-miR-432-5p	MIMAT0002814
hsa-miR-433-3p	MIMAT0001627
hsa-miR-433-5p	MIMAT0026554
hsa-miR-4421	MIMAT0018934
hsa-miR-4425	MIMAT0018940
hsa-miR-4431	MIMAT0018947
hsa-miR-4435	MIMAT0018951
hsa-miR-4443	MIMAT0018961
hsa-miR-4448	MIMAT0018967
hsa-miR-4451	MIMAT0018973
hsa-miR-4454+hsa-miR-7975	MIMAT0018976
hsa-miR-4455	MIMAT0018977
hsa-miR-4458	MIMAT0018980
hsa-miR-4461	MIMAT0018983
hsa-miR-448	MIMAT0001532
hsa-miR-4485-3p	MIMAT0019019
hsa-miR-4488	MIMAT0019022
hsa-miR-449a	MIMAT0001541
hsa-miR-449b-5p	MIMAT0003327
hsa-miR-449c-5p	MIMAT0010251
hsa-miR-450a-1-3p	MIMAT0022700
hsa-miR-450a-2-3p	MIMAT0031074
hsa-miR-450a-5p	MIMAT0001545
hsa-miR-450b-3p	MIMAT0004910
hsa-miR-450b-5p	MIMAT0004909
hsa-miR-4516	MIMAT0019053
hsa-miR-451a	MIMAT0001631
hsa-miR-452-5p	MIMAT0001635
hsa-miR-4521	MIMAT0019058
hsa-miR-4524a-5p	MIMAT0019062

hsa-miR-4531	MIMAT0019070
hsa-miR-4532	MIMAT0019071
hsa-miR-4536-3p	MIMAT0020959
hsa-miR-4536-5p	MIMAT0019078
hsa-miR-454-3p	MIMAT0003885
hsa-miR-455-3p	MIMAT0004784
hsa-miR-455-5p	MIMAT0003150
hsa-miR-4647	MIMAT0019709
hsa-miR-4707-3p	MIMAT0019808
hsa-miR-4707-5p	MIMAT0019807
hsa-miR-4741	MIMAT0019871
hsa-miR-4755-5p	MIMAT0019895
hsa-miR-4787-3p	MIMAT0019957
hsa-miR-4787-5p	MIMAT0019956
hsa-miR-4792	MIMAT0019964
hsa-miR-483-3p	MIMAT0002173
hsa-miR-483-5p	MIMAT0004761
hsa-miR-484	MIMAT0002174
hsa-miR-485-3p	MIMAT0002176
hsa-miR-485-5p	MIMAT0002175
hsa-miR-486-3p	MIMAT0004762
hsa-miR-487a-3p	MIMAT0002178
hsa-miR-487b-3p	MIMAT0003180
hsa-miR-487b-5p	MIMAT0026614
hsa-miR-488-3p	MIMAT0004763
hsa-miR-489-3p	MIMAT0002805
hsa-miR-490-3p	MIMAT0002806
hsa-miR-490-5p	MIMAT0004764
hsa-miR-491-3p	MIMAT0004765
hsa-miR-491-5p	MIMAT0002807
hsa-miR-492	MIMAT0002812
hsa-miR-493-3p	MIMAT0003161
hsa-miR-494-3p	MIMAT0002816
hsa-miR-494-5p	MIMAT0026607
hsa-miR-495-3p	MIMAT0002817
hsa-miR-495-5p	MIMAT0022924
hsa-miR-496	MIMAT0002818
hsa-miR-497-5p	MIMAT0002820
hsa-miR-498	MIMAT0002824
hsa-miR-499a-3p	MIMAT0004772
hsa-miR-499a-5p	MIMAT0002870
hsa-miR-499b-3p	MIMAT0019898
hsa-miR-499b-5p	MIMAT0019897
hsa-miR-5001-3p	MIMAT0021022
hsa-miR-5001-5p	MIMAT0021021
hsa-miR-500a-5p+hsa-miR-501-5p	MIMAT0004773
hsa-miR-501-3p	MIMAT0004774

hsa-miR-5010-3p	MIMAT0021044
hsa-miR-5010-5p	MIMAT0021043
hsa-miR-502-3p	MIMAT0004775
hsa-miR-502-5p	MIMAT0002873
hsa-miR-503-3p	MIMAT0022925
hsa-miR-503-5p	MIMAT0002874
hsa-miR-504-3p	MIMAT0026612
hsa-miR-504-5p	MIMAT0002875
hsa-miR-505-3p	MIMAT0002876
hsa-miR-506-3p	MIMAT0002878
hsa-miR-506-5p	MIMAT0022701
hsa-miR-507	MIMAT0002879
hsa-miR-508-3p	MIMAT0002880
hsa-miR-508-5p	MIMAT0004778
hsa-miR-509-3-5p	MIMAT0004975
hsa-miR-509-3p	MIMAT0002881
hsa-miR-509-5p	MIMAT0004779
hsa-miR-510-3p	MIMAT0026613
hsa-miR-510-5p	MIMAT0002882
hsa-miR-511-5p	MIMAT0002808
hsa-miR-512-3p	MIMAT0002823
hsa-miR-512-5p	MIMAT0002822
hsa-miR-513a-3p	MIMAT0004777
hsa-miR-513a-5p	MIMAT0002877
hsa-miR-513b-5p	MIMAT0005788
hsa-miR-513c-3p	MIMAT0022728
hsa-miR-513c-5p	MIMAT0005789
hsa-miR-514a-3p	MIMAT0002883
hsa-miR-514a-5p	MIMAT0022702
hsa-miR-514b-3p	MIMAT0015088
hsa-miR-514b-5p	MIMAT0015087
hsa-miR-515-3p	MIMAT0002827
hsa-miR-515-5p	MIMAT0002826
hsa-miR-516a-3p+hsa-miR-516b-3	MIMAT0006778
hsa-miR-516a-5p	MIMAT0004770
hsa-miR-516b-5p	MIMAT0002859
hsa-miR-517a-3p	MIMAT0002852
hsa-miR-517b-3p	MIMAT0002857
hsa-miR-517c-3p+hsa-miR-519a-3	MIMAT0002866
hsa-miR-518b	MIMAT0002844
hsa-miR-518c-3p	MIMAT0002848
hsa-miR-518d-3p	MIMAT0002864
hsa-miR-518e-3p	MIMAT0002861
hsa-miR-518f-3p	MIMAT0002842
hsa-miR-5196-3p+hsa-miR-6732-3	MIMAT0021129
hsa-miR-5196-5p	MIMAT0021128
hsa-miR-519b-3p	MIMAT0002837

hsa-miR-519b-5p+hsa-miR-519c-5	MIMAT0005454
hsa-miR-519c-3p	MIMAT0002832
hsa-miR-519d-3p	MIMAT0002853
hsa-miR-519e-3p	MIMAT0002829
hsa-miR-520a-3p	MIMAT0002834
hsa-miR-520a-5p	MIMAT0002833
hsa-miR-520b	MIMAT0002843
hsa-miR-520c-3p	MIMAT0002846
hsa-miR-520d-3p	MIMAT0002856
hsa-miR-520d-5p+hsa-miR-527+hs	MIMAT0002855
hsa-miR-520e	MIMAT0002825
hsa-miR-520f-3p	MIMAT0002830
hsa-miR-520g-3p	MIMAT0002858
hsa-miR-520h	MIMAT0002867
hsa-miR-521	MIMAT0002854
hsa-miR-522-3p	MIMAT0002868
hsa-miR-523-3p	MIMAT0002840
hsa-miR-524-3p	MIMAT0002850
hsa-miR-525-3p	MIMAT0002839
hsa-miR-525-5p	MIMAT0002838
hsa-miR-526a+hsa-miR-518c-5p+h	MIMAT0002845
hsa-miR-526b-5p	MIMAT0002835
hsa-miR-532-3p	MIMAT0004780
hsa-miR-532-5p	MIMAT0002888
hsa-miR-539-3p	MIMAT0022705
hsa-miR-539-5p	MIMAT0003163
hsa-miR-541-3p	MIMAT0004920
hsa-miR-542-3p	MIMAT0003389
hsa-miR-542-5p	MIMAT0003340
hsa-miR-543	MIMAT0004954
hsa-miR-544a	MIMAT0003164
hsa-miR-545-3p	MIMAT0003165
hsa-miR-548a-3p	MIMAT0003251
hsa-miR-548a-5p	MIMAT0004803
hsa-miR-548aa+hsa-miR-548t-3p	MIMAT0018447
hsa-miR-548ad-3p	MIMAT0018946
hsa-miR-548ah-5p	MIMAT0018972
hsa-miR-548ai+hsa-miR-570-5p	MIMAT0018989
hsa-miR-548ak	MIMAT0019013
hsa-miR-548al	MIMAT0019024
hsa-miR-548ar-3p	MIMAT0022266
hsa-miR-548ar-5p	MIMAT0022265
hsa-miR-548b-3p	MIMAT0003254
hsa-miR-548c-5p+hsa-miR-548o-5	MIMAT0004806
hsa-miR-548d-3p	MIMAT0003323
hsa-miR-548d-5p	MIMAT0004812
hsa-miR-548e-3p	MIMAT0005874

hsa-miR-548e-5p	MIMAT0026736
hsa-miR-548g-3p	MIMAT0005912
hsa-miR-548h-5p	MIMAT0005928
hsa-miR-548i	MIMAT0005935
hsa-miR-548j-3p	MIMAT0026737
hsa-miR-548j-5p	MIMAT0005875
hsa-miR-548k	MIMAT0005882
hsa-miR-548l	MIMAT0005889
hsa-miR-548m	MIMAT0005917
hsa-miR-548n	MIMAT0005916
hsa-miR-548o-3p+hsa-miR-548ah-	MIMAT0005919
hsa-miR-548q	MIMAT0011163
hsa-miR-548v	MIMAT0015020
hsa-miR-548y	MIMAT0018354
hsa-miR-548z+hsa-miR-548h-3p	MIMAT0018446
hsa-miR-549a	MIMAT0003333
hsa-miR-550a-5p	MIMAT0004800
hsa-miR-551a	MIMAT0003214
hsa-miR-551b-3p	MIMAT0003233
hsa-miR-552-3p	MIMAT0003215
hsa-miR-553	MIMAT0003216
hsa-miR-554	MIMAT0003217
hsa-miR-555	MIMAT0003219
hsa-miR-556-3p	MIMAT0004793
hsa-miR-556-5p	MIMAT0003220
hsa-miR-561-3p	MIMAT0003225
hsa-miR-561-5p	MIMAT0022706
hsa-miR-562	MIMAT0003226
hsa-miR-563	MIMAT0003227
hsa-miR-564	MIMAT0003228
hsa-miR-566	MIMAT0003230
hsa-miR-567	MIMAT0003231
hsa-miR-568	MIMAT0003232
hsa-miR-570-3p	MIMAT0003235
hsa-miR-571	MIMAT0003236
hsa-miR-572	MIMAT0003237
hsa-miR-573	MIMAT0003238
hsa-miR-574-3p	MIMAT0003239
hsa-miR-574-5p	MIMAT0004795
hsa-miR-575	MIMAT0003240
hsa-miR-576-3p	MIMAT0004796
hsa-miR-576-5p	MIMAT0003241
hsa-miR-577	MIMAT0003242
hsa-miR-578	MIMAT0003243
hsa-miR-579-3p	MIMAT0003244
hsa-miR-579-5p	MIMAT0026616
hsa-miR-580-3p	MIMAT0003245

hsa-miR-582-3p	MIMAT0004797
hsa-miR-582-5p	MIMAT0003247
hsa-miR-584-3p	MIMAT0022708
hsa-miR-584-5p	MIMAT0003249
hsa-miR-585-3p	MIMAT0003250
hsa-miR-587	MIMAT0003253
hsa-miR-589-5p	MIMAT0004799
hsa-miR-590-3p	MIMAT0004801
hsa-miR-590-5p	MIMAT0003258
hsa-miR-591	MIMAT0003259
hsa-miR-592	MIMAT0003260
hsa-miR-593-3p	MIMAT0004802
hsa-miR-595	MIMAT0003263
hsa-miR-596	MIMAT0003264
hsa-miR-597-5p	MIMAT0003265
hsa-miR-598-3p	MIMAT0003266
hsa-miR-599	MIMAT0003267
hsa-miR-600	MIMAT0003268
hsa-miR-601	MIMAT0003269
hsa-miR-603	MIMAT0003271
hsa-miR-604	MIMAT0003272
hsa-miR-605-5p	MIMAT0003273
hsa-miR-606	MIMAT0003274
hsa-miR-607	MIMAT0003275
hsa-miR-608	MIMAT0003276
hsa-miR-610	MIMAT0003278
hsa-miR-612	MIMAT0003280
hsa-miR-613	MIMAT0003281
hsa-miR-614	MIMAT0003282
hsa-miR-615-3p	MIMAT0003283
hsa-miR-615-5p	MIMAT0004804
hsa-miR-616-3p	MIMAT0004805
hsa-miR-617	MIMAT0003286
hsa-miR-619-3p	MIMAT0003288
hsa-miR-620	MIMAT0003289
hsa-miR-624-3p	MIMAT0004807
hsa-miR-625-5p	MIMAT0003294
hsa-miR-626	MIMAT0003295
hsa-miR-627-3p	MIMAT0026623
hsa-miR-627-5p	MIMAT0003296
hsa-miR-628-3p	MIMAT0003297
hsa-miR-628-5p	MIMAT0004809
hsa-miR-629-5p	MIMAT0004810
hsa-miR-630	MIMAT0003299
hsa-miR-631	MIMAT0003300
hsa-miR-637	MIMAT0003307
hsa-miR-638	MIMAT0003308

hsa-miR-639	MIMAT0003309
hsa-miR-640	MIMAT0003310
hsa-miR-641	MIMAT0003311
hsa-miR-642a-3p	MIMAT0020924
hsa-miR-642a-5p	MIMAT0003312
hsa-miR-643	MIMAT0003313
hsa-miR-644a	MIMAT0003314
hsa-miR-648	MIMAT0003318
hsa-miR-649	MIMAT0003319
hsa-miR-650	MIMAT0003320
hsa-miR-6503-3p	MIMAT0025463
hsa-miR-6503-5p	MIMAT0025462
hsa-miR-651-3p	MIMAT0026624
hsa-miR-651-5p	MIMAT0003321
hsa-miR-6511a-3p	MIMAT0025479
hsa-miR-6511a-5p	MIMAT0025478
hsa-miR-652-3p	MIMAT0003322
hsa-miR-652-5p	MIMAT0022709
hsa-miR-654-3p	MIMAT0004814
hsa-miR-654-5p	MIMAT0003330
hsa-miR-655-3p	MIMAT0003331
hsa-miR-656-3p	MIMAT0003332
hsa-miR-660-3p	MIMAT0022711
hsa-miR-660-5p	MIMAT0003338
hsa-miR-661	MIMAT0003324
hsa-miR-663a	MIMAT0003326
hsa-miR-664a-3p	MIMAT0005949
hsa-miR-664b-3p	MIMAT0022272
hsa-miR-664b-5p	MIMAT0022271
hsa-miR-665	MIMAT0004952
hsa-miR-671-3p	MIMAT0004819
hsa-miR-671-5p	MIMAT0003880
hsa-miR-6720-3p	MIMAT0025851
hsa-miR-6721-5p	MIMAT0025852
hsa-miR-6724-5p	MIMAT0025856
hsa-miR-675-5p	MIMAT0004284
hsa-miR-7-5p	MIMAT0000252
hsa-miR-708-5p	MIMAT0004926
hsa-miR-744-5p	MIMAT0004945
hsa-miR-758-3p+hsa-miR-411-3p	MIMAT0003879
hsa-miR-758-5p	MIMAT0022929
hsa-miR-760	MIMAT0004957
hsa-miR-761	MIMAT0010364
hsa-miR-764	MIMAT0010367
hsa-miR-765	MIMAT0003945
hsa-miR-766-3p	MIMAT0003888
hsa-miR-766-5p	MIMAT0022714

hsa-miR-767-3p	MIMAT0003883
hsa-miR-767-5p	MIMAT0003882
hsa-miR-769-3p	MIMAT0003887
hsa-miR-769-5p	MIMAT0003886
hsa-miR-770-5p	MIMAT0003948
hsa-miR-802	MIMAT0004185
hsa-miR-873-3p	MIMAT0022717
hsa-miR-873-5p	MIMAT0004953
hsa-miR-874-3p	MIMAT0004911
hsa-miR-874-5p	MIMAT0026718
hsa-miR-875-3p	MIMAT0004923
hsa-miR-876-3p	MIMAT0004925
hsa-miR-876-5p	MIMAT0004924
hsa-miR-877-5p	MIMAT0004949
hsa-miR-885-3p	MIMAT0004948
hsa-miR-885-5p	MIMAT0004947
hsa-miR-887-3p	MIMAT0004951
hsa-miR-887-5p	MIMAT0026720
hsa-miR-888-5p	MIMAT0004916
hsa-miR-889-3p	MIMAT0004921
hsa-miR-890	MIMAT0004912
hsa-miR-891a-5p	MIMAT0004902
hsa-miR-891b	MIMAT0004913
hsa-miR-892a	MIMAT0004907
hsa-miR-892b	MIMAT0004918
hsa-miR-9-5p	MIMAT0000441
hsa-miR-922	MIMAT0004972
hsa-miR-924	MIMAT0004974
hsa-miR-92a-1-5p	MIMAT0004507
hsa-miR-92a-3p	MIMAT0000092
hsa-miR-92b-3p	MIMAT0003218
hsa-miR-93-5p	MIMAT0000093
hsa-miR-933	MIMAT0004976
hsa-miR-934	MIMAT0004977
hsa-miR-935	MIMAT0004978
hsa-miR-936	MIMAT0004979
hsa-miR-937-3p	MIMAT0004980
hsa-miR-939-5p	MIMAT0004982
hsa-miR-940	MIMAT0004983
hsa-miR-941	MIMAT0004984
hsa-miR-942-3p	MIMAT0026734
hsa-miR-942-5p	MIMAT0004985
hsa-miR-944	MIMAT0004987
hsa-miR-95-3p	MIMAT0000094
hsa-miR-96-5p	MIMAT0000095
hsa-miR-98-3p	MIMAT0022842
hsa-miR-98-5p	MIMAT0000096

hsa-miR-99a-5p	MIMAT0000097
hsa-miR-99b-5p	MIMAT0000689
