# Supplementary material for: Inflammatory Changes after Medical Suppression of Suspected Endometriosis for Implantation Failure: Preliminary Results
Source: Int J Mol Sci. 2024 Jun 22;25(13):6852. doi: 10.3390/ijms25136852 (PMC11241468; doi:10.3390/ijms25136852)
Supplement: Supplementary file 1 [file ijms-25-06852-s001.zip › Supplementary Table S5.pdf]

Table S5. miRNA differences in pre- and post-treatment comparison following treatment with OCPs.

Symbol	Accession	p-value	fold change
hsa-miR-2110	MIMAT0010133	0.046193084	2.065357276
hsa-miR-1293	MIMAT0005883	0.039035994	2.264418799
hsa-miR-25-3p	MIMAT0000081	0.008868135	1.558769128
hsa-miR-1301-3p	MIMAT0005797	0.036940255	2.264625002
hsa-miR-204-5p	MIMAT0000265	0.03767839	1.734822217
hsa-miR-874-5p	MIMAT0026718	0.043723737	0.61616545
hsa-miR-497-5p	MIMAT0002820	0.02459794	0.454297039
hsa-miR-539-5p	MIMAT0003163	0.007542426	0.595962646
hsa-miR-514a-5p	MIMAT0022702	0.034330881	0.59648782
hsa-miR-3690	MIMAT0018119	0.007051391	0.667292279
