# Supplementary material for: Inflammatory Changes after Medical Suppression of Suspected Endometriosis for Implantation Failure: Preliminary Results
Source: Int J Mol Sci. 2024 Jun 22;25(13):6852. doi: 10.3390/ijms25136852 (PMC11241468; doi:10.3390/ijms25136852)
Supplement: Supplementary file 1 [file ijms-25-06852-s001.zip › Supplementary Table S6.pdf]

Table S6. miRNA differences in pre- and post-treatment comparison following treatment with elagolix.

Symbol	Accession	p-value	fold change
hsa-miR-574-3p	MIMAT0003239	0.025069099	1.56571764
hsa-miR-1306-5p	MIMAT0022726	0.009353113	1.698479823
hsa-miR-532-5p	MIMAT0002888	0.003160429	1.765897723
hsa-miR-128-2-5p	MIMAT0031095	0.000499245	4.204234519
hsa-miR-744-5p	MIMAT0004945	0.021626114	1.897616233
hsa-miR-1253	MIMAT0005904	0.006671468	2.449749526
hsa-miR-139-5p	MIMAT0000250	0.023753309	2.44501521
hsa-miR-548m	MIMAT0005917	0.008163544	2.86141596
hsa-miR-548e-5p	MIMAT0026736	0.037187412	2.205303024
hsa-miR-219a-5p	MIMAT0000276	0.043185546	1.932711359
hsa-miR-483-3p	MIMAT0002173	0.002653934	2.80339236
hsa-miR-1205	MIMAT0005869	0.001686271	4.697489586
hsa-miR-337-5p	MIMAT0004695	0.009249259	2.153424094
hsa-miR-1185-5p	MIMAT0005798	0.018445408	2.121343812
hsa-miR-548v	MIMAT0015020	0.015653487	2.138722148
hsa-miR-1296-5p	MIMAT0005794	0.015590717	1.948930205
hsa-miR-324-3p	MIMAT0000762	0.00719898	2.005701455
hsa-miR-1281	MIMAT0005939	0.005204654	3.194871717
hsa-miR-4286	MIMAT0016916	0.007589101	2.751389473
hsa-miR-3605-5p	MIMAT0017981	0.012890071	4.254752135
hsa-miR-3168	MIMAT0015043	0.011711184	2.560867466
hsa-miR-1245a	MIMAT0005897	0.045071745	2.246466602
hsa-miR-663a	MIMAT0003326	0.00226717	2.753914855
hsa-miR-153-3p	MIMAT0000439	0.009531163	2.031725749
hsa-miR-3916	MIMAT0018190	0.002125141	2.76425474
hsa-miR-301a-5p	MIMAT0022696	0.009523411	1.875922651
hsa-miR-133b	MIMAT0000770	0.01563472	2.124131809
hsa-miR-651-3p	MIMAT0026624	0.012479852	2.801663908
hsa-miR-617	MIMAT0003286	0.005828719	1.956070288
hsa-miR-1297	MIMAT0005886	0.016215944	2.042422625
hsa-miR-571	MIMAT0003236	0.002762565	2.002675516
hsa-miR-504-3p	MIMAT0026612	0.003290541	2.045958092
hsa-miR-499a-3p	MIMAT0004772	0.00175638	3.175603019
hsa-miR-548j-5p	MIMAT0005875	0.00192424	2.344557269
hsa-miR-325	MIMAT0000771	0.025747521	2.071562547
hsa-miR-3130-3p	MIMAT0014994	0.007916941	2.402298231
hsa-miR-95-3p	MIMAT0000094	0.002880601	2.466574038

hsa-miR-181b-2-3p	MIMAT0031893	0.010395757	2.188355749
hsa-miR-1249-5p	MIMAT0032029	0.013172933	2.308688562
hsa-miR-211-5p	MIMAT0000268	0.014369356	2.566015231
hsa-miR-219b-3p	MIMAT0019748	0.011010822	2.059974871
hsa-miR-655-3p	MIMAT0003331	0.00022825	2.589173633
hsa-miR-513a-3p	MIMAT0004777	0.002102879	2.614965125
hsa-miR-382-3p	MIMAT0022697	0.0029489	2.435229694
hsa-miR-3180	MIMAT0018178	0.046602597	3.099879045
hsa-miR-25-5p	MIMAT0004498	0.021328252	3.000778692
hsa-miR-802	MIMAT0004185	0.015832017	2.439732169
hsa-miR-1185-2-3p	MIMAT0022713	0.037428319	2.010530009
hsa-miR-551b-3p	MIMAT0003233	0.042790265	1.842562135
hsa-miR-5196-5p	MIMAT0021128	0.015792465	3.140502664
hsa-miR-342-5p	MIMAT0004694	0.030289903	1.466430602
hsa-miR-3613-3p	MIMAT0017991	0.04845925	2.79085246
hsa-miR-135a-5p	MIMAT0000428	0.030504377	2.397678204
hsa-miR-133a-3p	MIMAT0000427	0.020112892	2.191409988
hsa-miR-3202	MIMAT0015089	0.021990836	1.774895313
hsa-miR-302a-3p	MIMAT0000684	0.010845574	2.396095754
hsa-miR-3179	MIMAT0015056	0.029736651	1.761269817
hsa-miR-593-3p	MIMAT0004802	0.021576994	2.427803272
hsa-miR-147b	MIMAT0004928	0.036311937	2.244477031
hsa-miR-1269a	MIMAT0005923	0.01830783	2.07343739
hsa-miR-767-5p	MIMAT0003882	0.026260286	1.863643778
hsa-miR-561-3p	MIMAT0003225	0.006293736	2.105354336
hsa-miR-1245b-3p	MIMAT0019951	0.012264457	2.085938814
hsa-miR-891b	MIMAT0004913	0.038865433	2.188802608
hsa-miR-605-5p	MIMAT0003273	0.021725891	2.483763032
hsa-miR-4488	MIMAT0019022	0.019926259	2.455936047
hsa-miR-1908-3p	MIMAT0026916	0.046621046	1.54547648
hsa-miR-626	MIMAT0003295	0.025580196	2.391488841
hsa-miR-519d-3p	MIMAT0002853	0.023024327	2.20986368
hsa-miR-133a-5p	MIMAT0026478	0.013292003	3.090691053
hsa-miR-608	MIMAT0003276	0.030533047	2.032548195
hsa-miR-887-5p	MIMAT0026720	0.026601613	3.010734121
hsa-miR-128-1-5p	MIMAT0026477	0.023578087	1.774156966
hsa-miR-1910-3p	MIMAT0026917	0.039524523	1.75392238
hsa-miR-208a-3p	MIMAT0000241	0.014002945	2.552115515
hsa-miR-149-5p	MIMAT0000450	0.02237245	2.103943782
hsa-miR-1972	MIMAT0009447	0.007365229	2.370306978
hsa-miR-98-3p	MIMAT0022842	0.018833882	1.669794721
hsa-miR-520h	MIMAT0002867	0.042358384	2.273206869

hsa-miR-489-3p	MIMAT0002805	0.009223416	4.135993694
hsa-miR-378h	MIMAT0018984	0.004024541	2.168022513
hsa-miR-548h-5p	MIMAT0005928	0.020613174	4.164728741
hsa-miR-519c-3p	MIMAT0002832	0.01761044	2.509042441
hsa-miR-129-5p	MIMAT0000242	0.019298985	3.034989163
hsa-miR-125a-3p	MIMAT0004602	0.009049357	2.493802298
hsa-miR-6721-5p	MIMAT0025852	0.015797963	2.963675437
hsa-miR-589-5p	MIMAT0004799	0.037112359	3.172706147
hsa-miR-1279	MIMAT0005937	0.048899139	3.405024989
hsa-miR-1271-3p	MIMAT0022712	0.034227524	1.819937441
hsa-miR-580-3p	MIMAT0003245	0.042761214	1.749807552
hsa-miR-513c-5p	MIMAT0005789	0.016236089	2.610865108
hsa-miR-525-5p	MIMAT0002838	0.009219067	2.807806316
hsa-miR-374a-3p	MIMAT0004688	0.004894609	4.565340499
hsa-miR-642a-5p	MIMAT0003312	0.036049638	2.638848967
hsa-miR-134-5p?miR-6728-5p	MIMAT0000447	0.013398879	3.789404327
hsa-miR-99b-5p	MIMAT0000689	0.023341897	2.246411472
hsa-miR-615-3p	MIMAT0003283	0.008666679	3.079752249
hsa-miR-198	MIMAT0000228	0.024325585	1.960139005
hsa-miR-381-5p	MIMAT0022862	0.045527415	1.932597976
hsa-miR-654-3p	MIMAT0004814	0.027371699	1.631119142
hsa-miR-1276	MIMAT0005930	0.044868828	1.561262185
hsa-miR-1908-5p	MIMAT0007881	0.042453593	1.778625462
hsa-miR-766-5p	MIMAT0022714	0.015271296	3.551203378
hsa-miR-378e	MIMAT0018927	0.03182035	2.323990791
hsa-miR-548ar-3p	MIMAT0022266	0.022905373	2.627681402
hsa-miR-433-3p	MIMAT0001627	0.041695482	2.274323254
hsa-miR-210-5p	MIMAT0026475	0.029521123	1.813374959
hsa-miR-1197	MIMAT0005955	0.019384296	2.444249028
hsa-miR-346	MIMAT0000773	0.030291277	2.309621596
hsa-miR-1250-5p	MIMAT0005902	0.038334344	3.642615849
hsa-miR-892b	MIMAT0004918	0.021670643	2.757123198
hsa-miR-1273c	MIMAT0015017	0.022994699	3.400099483
hsa-miR-628-5p	MIMAT0004809	0.034275391	3.398426564
hsa-miR-3144-3p	MIMAT0015015	0.037021811	2.064839901
hsa-miR-661	MIMAT0003324	0.011586549	3.830594168
hsa-miR-656-3p	MIMAT0003332	0.02131492	4.94233468
hsa-miR-9-5p	MIMAT0000441	0.012553304	3.691394388
hsa-miR-549a	MIMAT0003333	0.03290357	3.17093916
hsa-miR-323a-5p	MIMAT0004696	0.039310608	2.322187749
hsa-miR-138-5p	MIMAT0000430	0.038040116	2.456456828
hsa-miR-1277-3p	MIMAT0005933	0.041072323	1.870873991

hsa-miR-1200	MIMAT0005863	0.043956802	2.148948488
hsa-miR-1178-3p	MIMAT0005823	0.026657194	3.706051175
hsa-miR-574-5p	MIMAT0004795	0.034625889	2.336465224
hsa-miR-323a-3p	MIMAT0000755	0.049744082	2.186021125
hsa-miR-516a-5p	MIMAT0004770	0.022788769	2.190167802
hsa-miR-548ad-3p	MIMAT0018946	0.04854946	3.07409022
hsa-miR-939-5p	MIMAT0004982	0.007481515	3.039551768
hsa-miR-4532	MIMAT0019071	0.003351726	3.755373828
hsa-miR-564	MIMAT0003228	0.005864115	3.319452595
hsa-miR-2117	MIMAT0011162	0.017587392	1.973605104
hsa-miR-526b-5p	MIMAT0002835	0.011405043	2.892948816
hsa-miR-320a	MIMAT0000510	0.015051904	2.573559044
hsa-miR-509-3-5p	MIMAT0004975	0.012562939	3.796857881
hsa-miR-224-5p	MIMAT0000281	0.005934357	2.343101454
hsa-miR-877-5p	MIMAT0004949	0.005434544	3.019911983
hsa-miR-10b-5p	MIMAT0000254	0.003085411	3.395671525
hsa-miR-328-3p	MIMAT0000752	0.012210754	1.7990609
hsa-miR-1296-3p	MIMAT0026637	0.027641449	2.127437241
hsa-miR-214-3p	MIMAT0000271	0.03922433	2.325449527
hsa-miR-548q	MIMAT0011163	0.010645191	3.463267747
hsa-miR-302f	MIMAT0005932	0.038441229	2.169268809
hsa-miR-208b-3p	MIMAT0004960	0.030246842	2.235890555
hsa-miR-873-3p	MIMAT0022717	0.03207419	2.243934255
hsa-miR-1287-5p	MIMAT0005878	0.039970459	2.315516807
hsa-miR-944	MIMAT0004987	0.006016758	2.345051138
hsa-miR-181d-3p	MIMAT0026608	0.000142407	3.715862257
hsa-miR-296-3p	MIMAT0004679	0.000646393	2.471025839
hsa-miR-1204	MIMAT0005868	0.000974186	2.879762503
hsa-miR-671-5p	MIMAT0003880	0.004064464	3.886215535
hsa-miR-203a-5p	MIMAT0031890	0.00072126	2.444101395
hsa-miR-101-3p	MIMAT0000099	0.002186503	3.31486999
hsa-miR-152-3p	MIMAT0000438	0.000998384	3.278611176
hsa-miR-940	MIMAT0004983	0.005055708	1.76481478
hsa-miR-604	MIMAT0003272	0.035989365	2.642973711
hsa-miR-542-3p	MIMAT0003389	0.036797394	4.225873614
hsa-miR-455-5p	MIMAT0003150	0.018654892	2.154524379
hsa-miR-1307-3p	MIMAT0005951	0.001847951	2.139161877
hsa-miR-449a	MIMAT0001541	0.006501809	3.189059179
hsa-miR-190a-3p	MIMAT0026482	0.015781349	3.017046696
hsa-miR-1287-3p	MIMAT0026738	0.008422305	3.353446185
hsa-miR-1252-5p	MIMAT0005944	0.003546199	2.554764068
hsa-miR-1302	MIMAT0005890	0.037293284	2.248242618

hsa-miR-372-3p	MIMAT0000724	0.010214777	3.530145237
hsa-miR-18b-5p	MIMAT0001412	0.00680801	3.850685581
hsa-miR-708-5p	MIMAT0004926	0.012397231	2.363215815
hsa-miR-606	MIMAT0003274	0.003501203	1.982444679
hsa-miR-1260b	MIMAT0015041	0.044692322	3.071090547
hsa-miR-134-3p	MIMAT0026481	0.002548568	2.774628174
hsa-miR-590-5p	MIMAT0003258	0.000937545	2.640362865
hsa-miR-2113	MIMAT0009206	0.002643756	4.431117999
hsa-miR-363-5p	MIMAT0003385	0.016854982	1.886257878
hsa-miR-614	MIMAT0003282	0.029558334	2.696872002
hsa-miR-2682-5p	MIMAT0013517	0.022395335	3.170723531
hsa-miR-551a	MIMAT0003214	0.009481983	1.824076783
hsa-miR-202-3p	MIMAT0002811	0.01179548	1.766829274
hsa-miR-1303	MIMAT0005891	0.018772975	1.961725288
hsa-miR-1-3p	MIMAT0000416	0.014250718	2.024458967
hsa-miR-1261	MIMAT0005913	0.020935667	2.670033492
hsa-miR-3158-3p	MIMAT0015032	0.033646469	2.068164656
hsa-miR-3605-3p	MIMAT0017982	0.029480769	2.70789302
hsa-miR-323b-5p	MIMAT0001630	0.02008304	2.773141092
hsa-miR-335-5p	MIMAT0000765	0.008113927	3.266746778
hsa-miR-152-5p	MIMAT0026479	0.007776446	2.360480863
hsa-miR-519b-5p?miR-519c-5p?miR-523-5p?miR-518e-5p?miR-522-5p?miR-519a-5p	MIMAT0005454	0.024977923	3.190325336
hsa-miR-631	MIMAT0003300	0.004458903	3.471831317
hsa-miR-1226-3p	MIMAT0005577	0.010553226	4.277997118
hsa-miR-4435	MIMAT0018951	0.012771181	1.697618816
hsa-miR-206	MIMAT0000462	0.048046051	3.015168078
hsa-miR-1295a	MIMAT0005885	0.027296061	3.096451971
hsa-miR-365a-3p?miR-365b-3p	MIMAT0000710	0.034054445	2.120664688
hsa-miR-216a-5p	MIMAT0000273	0.038141419	2.718039224
hsa-miR-495-3p	MIMAT0002817	0.020778587	2.945557228
hsa-miR-302d-3p	MIMAT0000718	0.017521043	1.812089951
hsa-miR-590-3p	MIMAT0004801	0.037959399	2.820002701
hsa-miR-3934-5p	MIMAT0018349	0.032350878	2.564936231
hsa-miR-10a-5p	MIMAT0000253	0.020555065	2.75842732
hsa-miR-548ai?miR-570-5p	MIMAT0018989	0.039916095	3.364220239
hsa-miR-4647	MIMAT0019709	0.021148798	3.338234256
hsa-miR-4425	MIMAT0018940	0.025360947	2.281964369
hsa-miR-339-3p	MIMAT0004702	0.018455704	3.802474082

hsa-miR-664b-5p	MIMAT0022271	0.0341992	2.634617672
hsa-miR-649	MIMAT0003319	0.023468692	2.497856766
hsa-miR-6511a-5p	MIMAT0025478	0.006627156	2.891916334
hsa-miR-520b	MIMAT0002843	0.00265166	3.802596935
hsa-miR-545-3p	MIMAT0003165	0.014171662	2.180297782
hsa-miR-2278	MIMAT0011778	0.032188398	2.761594368
hsa-miR-598-3p	MIMAT0003266	0.018013194	2.124290482
hsa-miR-1278	MIMAT0005936	0.031550289	2.33739326
hsa-miR-3613-5p	MIMAT0017990	0.008019583	2.54718358
hsa-miR-518e-3p	MIMAT0002861	0.040696007	2.918218069
hsa-miR-196a-3p	MIMAT0004562	0.028729603	2.930126206
hsa-miR-766-3p	MIMAT0003888	0.013942201	2.532462046
hsa-miR-572	MIMAT0003237	0.016832096	3.410918298
hsa-miR-671-3p	MIMAT0004819	0.041107789	2.269734405
hsa-miR-200b-3p	MIMAT0000318	0.030792627	2.408181646
hsa-miR-432-5p	MIMAT0002814	0.027424568	2.105334504
hsa-miR-429	MIMAT0001536	0.034207366	1.656259532
hsa-miR-4458	MIMAT0018980	0.004140946	2.417815721
hsa-miR-1323	MIMAT0005795	0.015818348	2.534768472
hsa-miR-33b-5p	MIMAT0003301	0.039075308	2.257126992
hsa-miR-1289	MIMAT0005879	0.019083481	3.859717848
hsa-miR-770-5p	MIMAT0003948	0.043924595	2.724057264
hsa-miR-4524a-5p	MIMAT0019062	0.035551998	2.09194057
hsa-miR-552-3p	MIMAT0003215	0.035573291	2.170428301
hsa-miR-630	MIMAT0003299	0.008552065	2.543572746
hsa-miR-601	MIMAT0003269	0.004854484	2.274290179
hsa-miR-876-5p	MIMAT0004924	0.024414991	2.349512876
hsa-miR-4536-5p	MIMAT0019078	0.02171953	3.013320259
hsa-miR-654-5p	MIMAT0003330	0.015347158	2.717574586
hsa-miR-205-5p	MIMAT0000266	0.028954659	3.68772623
hsa-miR-373-3p	MIMAT0000726	0.034827239	2.764494352
hsa-miR-1909-3p	MIMAT0007883	0.043872536	2.198751462
hsa-miR-1286	MIMAT0005877	0.011152816	2.662685378
hsa-miR-1255b-5p	MIMAT0005945	0.006720702	2.471016651
hsa-miR-541-3p	MIMAT0004920	0.000526382	5.080329484
hsa-miR-136-5p	MIMAT0000448	0.001608986	2.344392992
hsa-miR-769-5p	MIMAT0003886	0.018654559	2.453604845
hsa-miR-1245b-5p	MIMAT0019950	0.01594907	3.09424112
hsa-miR-582-5p	MIMAT0003247	0.025336441	6.056787114
hsa-miR-1290	MIMAT0005880	0.010548552	2.532118052
hsa-miR-182-3p	MIMAT0000260	0.026033937	2.403782086
hsa-miR-1285-5p	MIMAT0022719	0.03387028	1.911002846

hsa-miR-302c-3p	MIMAT0000717	0.017186969	2.550253489
hsa-miR-127-3p	MIMAT0000446	0.009356214	2.546121125
hsa-miR-507	MIMAT0002879	0.00419405	5.085582875
hsa-miR-122-5p	MIMAT0000421	0.009856335	1.858578328
hsa-miR-197-5p	MIMAT0022691	0.010960951	3.584886416
hsa-miR-548b-3p	MIMAT0003254	0.017354134	3.220278109
hsa-miR-1301-3p	MIMAT0005797	0.03145236	3.133946283
hsa-miR-548k	MIMAT0005882	0.013713785	4.977507496
hsa-miR-758-3p?miR-411-3p	MIMAT0003879	0.002559474	3.523522701
hsa-miR-135b-5p	MIMAT0000758	0.005454153	4.679042897
hsa-miR-514b-5p	MIMAT0015087	0.006532537	3.143805738
hsa-miR-483-5p	MIMAT0004761	0.011387245	2.708603302
hsa-miR-382-5p	MIMAT0000737	0.006893943	3.194305507
hsa-miR-1304-3p	MIMAT0022720	0.004435883	2.916617638
hsa-miR-563	MIMAT0003227	0.009354558	2.639905398
hsa-miR-137	MIMAT0000429	0.002191897	3.404684003
hsa-miR-643	MIMAT0003313	0.029046972	2.546596849
hsa-miR-190b	MIMAT0004929	0.017802843	3.160396277
hsa-miR-520d-3p	MIMAT0002856	0.021413798	3.594525537
hsa-miR-195-5p	MIMAT0000461	0.011382597	2.114571961
hsa-miR-410-3p	MIMAT0002171	0.038189983	2.04861248
hsa-miR-495-5p	MIMAT0022924	0.036249918	1.738733344
hsa-miR-23c	MIMAT0018000	0.015325034	2.49053712
hsa-miR-4792	MIMAT0019964	0.045432818	2.119636865
hsa-let-7e-5p	MIMAT0000066	0.020379964	2.102191603
hsa-miR-92b-3p	MIMAT0003218	0.02517393	2.355509907
hsa-miR-1254	MIMAT0005905	0.017883668	2.259314455
hsa-miR-539-3p	MIMAT0022705	0.04341398	1.712237939
hsa-miR-629-5p	MIMAT0004810	0.029453162	2.255486713
hsa-miR-3185	MIMAT0015065	0.030821808	2.1392294
hsa-miR-4461	MIMAT0018983	0.021552695	3.69100185
hsa-miR-297	MIMAT0004450	0.033083183	3.623587609
hsa-miR-1915-3p	MIMAT0007892	0.014634382	4.64375538
hsa-miR-203a-3p	MIMAT0000264	0.016511411	1.934317562
hsa-miR-520c-3p	MIMAT0002846	0.040260997	2.156646086
hsa-miR-1537-3p	MIMAT0007399	0.041767509	2.203950613
hsa-miR-450a-5p	MIMAT0001545	0.040555871	1.879745286
hsa-miR-3065-5p	MIMAT0015066	0.042959462	1.86436668
hsa-miR-888-5p	MIMAT0004916	0.013828484	1.773558215
hsa-miR-1268b	MIMAT0018925	0.018097494	2.502095209
hsa-miR-501-3p	MIMAT0004774	0.007228178	2.133079106
hsa-miR-421	MIMAT0003339	0.020712031	2.294971243

hsa-miR-34c-3p	MIMAT0004677	0.027604915	2.632563193
hsa-miR-1973	MIMAT0009448	0.038729403	2.446006573
hsa-miR-3151-5p	MIMAT0015024	0.024608193	3.52934171
hsa-miR-591	MIMAT0003259	0.010103035	2.550085335
hsa-miR-369-3p	MIMAT0000721	0.005382236	3.088645606
hsa-miR-1228-3p	MIMAT0005583	0.005309436	2.174456819
hsa-miR-511-5p	MIMAT0002808	0.027813826	2.288959815
hsa-miR-4707-3p	MIMAT0019808	0.038909731	2.634008843
hsa-miR-3192-5p	MIMAT0015076	0.016188179	1.760368762
hsa-miR-371a-5p	MIMAT0004687	0.002824143	2.035532816
hsa-miR-1293	MIMAT0005883	0.000819315	4.073105004
hsa-miR-627-3p	MIMAT0026623	0.045405802	1.852924458
hsa-miR-339-5p	MIMAT0000764	0.039168853	2.098497059
hsa-miR-503-5p	MIMAT0002874	0.010953058	2.145003562
hsa-miR-521	MIMAT0002854	0.048039249	4.46598739
hsa-miR-103a-3p	MIMAT0000101	0.012866975	2.783949489
hsa-miR-874-3p	MIMAT0004911	0.037627993	3.567390203
hsa-miR-624-3p	MIMAT0004807	0.031438321	2.749613563
hsa-miR-378g	MIMAT0018937	0.001570672	1.694380748
hsa-miR-3140-3p	MIMAT0015008	0.024363974	2.156406238
hsa-miR-1193	MIMAT0015049	0.041199507	2.226697099
hsa-miR-1206	MIMAT0005870	0.012617722	3.232788632
hsa-miR-941	MIMAT0004984	0.034414162	2.370627227
hsa-miR-548ak	MIMAT0019013	0.013475738	2.400212297
hsa-miR-520f-3p	MIMAT0002830	0.004081655	1.54200682
hsa-miR-200a-3p	MIMAT0000682	0.016350963	1.781987053
hsa-miR-34c-5p	MIMAT0000686	0.022688096	2.038170647
hsa-miR-6503-5p	MIMAT0025462	0.00809161	2.286394869
hsa-miR-512-3p	MIMAT0002823	0.002546982	4.782988748
hsa-miR-490-3p	MIMAT0002806	0.000690042	5.334061895
hsa-miR-524-3p	MIMAT0002850	0.014388342	1.921636377
hsa-miR-302a-5p	MIMAT0000683	0.002342749	3.970881084
hsa-miR-139-3p	MIMAT0004552	0.04016157	1.958147275
hsa-miR-510-5p	MIMAT0002882	0.011615701	2.188866452
hsa-miR-18a-5p	MIMAT0000072	0.023009917	2.211998575
hsa-miR-520e	MIMAT0002825	0.036185662	2.316179095
hsa-miR-378i	MIMAT0019074	0.018869205	1.532596339
hsa-miR-485-5p	MIMAT0002175	0.029368877	2.027470575
hsa-miR-320e	MIMAT0015072	0.021377716	1.474730069
hsa-miR-31-5p	MIMAT0000089	0.005559228	1.803805843
hsa-miR-219a-2-3p	MIMAT0004675	0.001989025	2.125728202
hsa-miR-518c-3p	MIMAT0002848	0.008964852	2.104529732

hsa-miR-142-5p	MIMAT0000433	0.027878902	1.632795571
hsa-miR-518b	MIMAT0002844	0.017753994	1.724747369
hsa-miR-3065-3p	MIMAT0015378	0.003184343	1.785408844
hsa-miR-4521	MIMAT0019058	0.038822831	1.692715985
hsa-miR-936	MIMAT0004979	0.009347696	2.017278015
hsa-miR-140-5p	MIMAT0000431	0.038590045	1.562745237
hsa-miR-532-3p	MIMAT0004780	0.017982991	1.547651242
hsa-miR-513c-3p	MIMAT0022728	0.016619701	1.451007899
hsa-miR-16-5p	MIMAT0000069	0.017366381	0.467485821
hsa-miR-142-3p	MIMAT0000434	0.02060626	0.405314483
